# Sleep-Disordered Breathing and Its Association with Nocturnal Enuresis at the Primary Schools in Saudi Arabia: A Cross-Sectional Study

**DOI:** 10.3390/children10061074

**Published:** 2023-06-18

**Authors:** Ali Abdullah Alshehri, Mohamed Soliman Hassan Zaki, Sameh Osama Nour, Wala H. Gadi, Basem A. Zogel, Samar M. Alfaifi, Enas M. Masmali, Amani B. Aburasain, Mohamed Osama Nour

**Affiliations:** 1 Department of Surgery, College of Medicine, Najran University, P.O. Box 1988, Najran 11001, Saudi Arabia; 2 Department of Chest Diseases, Faculty of Medicine, Al-Azhar University, Cairo 11651, Egypt; medochest@gmail.com (M.S.H.Z.); drsamehnour@gmail.com (S.O.N.); 3 College of Medicine, Jazan University, P.O. Box 114, Jazan 45142, Saudi Arabia; whalgadhi@gmail.com (W.H.G.); basem14201@hotmail.com (B.A.Z.); samaralfaifi1@gmail.com (S.M.A.); enas.m.masmali@gmail.com (E.M.M.); amaniaburasain@gmail.com (A.B.A.); 4 Department of Public Health and Community Medicine, Damietta Faculty of Medicine, Al-Azhar University, Damietta 34511, Egypt; monour@uqu.edu.sa; 5 Faculty of Public Health and Health Informatics, Umm Al-Qura University, P.O. Box 715, Makkah 21955, Saudi Arabia

**Keywords:** sleep-disordered breathing, nocturnal enuresis, primary school children, Saudi Arabia

## Abstract

The correlation between nocturnal enuresis (NE) and sleep-disordered breathing (SDB) was reported. We aim to determine whether there is an association between NE and SDB in children and to assess the prevalence of SDB and NE in primary school children aged 6–12 years in Saudi Arabia. A cross-sectional observational study was conducted among the caregivers of children aged 6–12 years in all Saudi Arabia regions. The data were gathered through a self-administered online questionnaire. It included demographic information, weight and height, and associated comorbidities, in addition to the weekly frequencies of snoring symptoms and of enuresis, as well as of unrefreshing sleep using Likert-type response scales. Counts and percentages, the mean ± standard deviation, chi-square test, independent samples *t*-test, and regression analysis were used in the statistical analysis using R v 3.6.3. The questionnaire was completed by 686 respondents. Most respondents did not report any comorbidities in their children (77.1%). Asthma and adenotonsillar hypertrophy were reported in 16.2% and 15.6% of children, respectively. Unrefreshing sleep, mouth breathing at night, snoring, chronic nasal obstruction, and difficulty breathing while asleep were reported once or twice per week in 38%, 34%, 28%, 18%, and 18% of children, respectively. The prevalence of NE was 22.3%, with about 36.6% of children having NE two or more times per week. Significantly, NE was reported in 26.6% of children who slept before 10 PM compared to 19% of children who slept after 10 PM; in 28.6% of children who snored or loudly snored (57.1%) three times or more per week; and in 51.2% and 27.5% of children with difficulty breathing while asleep and who breathed through their mouth at night for one or two nights per week, respectively. A multivariable regression analysis showed that male gender (OR = 1.52, *p* = 0.010), obesity (OR = 1.24, *p* = 0.028), early sleeping time (OR = 1.40, *p* = 0.048), loud snoring for three or more nights per week (OR = 1.54, *p* = 0.001), difficulty breathing for one or two nights per week (OR = 1.85, *p* = 0.010), and mouth breathing at night for one or two nights per week (OR = 1.55, *p* = 0.049) were associated with higher odds of NE. Our study revealed that 22.3% of primary school children reported suffering from NE. SDB is a common problem among children with NE. The exact mechanism that links SDB to the increase in the risk of NE is unknown. Male gender, obesity, early sleeping time, loud snoring, difficulty breathing, and mouth breathing at night are potential independent risk factors of NE in school-age children.

## 1. Introduction

Nocturnal enuresis (NE) or nighttime bedwetting is defined as the unintentional release of urine at night in children older than five according to the International Children’s Continence Society criteria [1]. It is a common problem in the developmental age that affects 15% of children who are five years old and might cause a lot of stress for the family. Primary and secondary enuresis are two different types of enuresis, in which primary enuresis represents urinary incontinence in a child who has never been dry, while secondary enuresis represents urinary incontinence in a child who has been dry for at least six months [2]. NE is classified as either monosymptomatic (when the only symptom is the release of urine during sleep) or non-monosymptomatic depending on whether daytime urine symptoms are present such as urgency, hesitancy, frequency, postponement, dyscoordination, urge incontinence, dribbling, or pain [1]. NE has a multifactorial etiology and may be fundamentally influenced by sleep-disordered breathing (SDB) [3].

SDB is a disease that varies from simple snoring to obstructive sleep apnea (OSA), and it is associated with the secondary cause of enuresis [4]. Primary snoring, upper airway resistance, obstructive hypoventilation, and OSA all represent the severity of obstructive SDB [5]. The most prevalent clinical symptom of OSA is habitual snoring. Although frequent snoring has consistently been linked to the existence of NE in population-based research, it is uncertain whether enuresis prevalence and SDB severity are related. One of the morbidities linked with SDB is monosymptomatic primary NE, and when it is treated with an adenotonsillectomy or other therapeutic measures such as nasal corticosteroids or rapid maxillary expansion, the enuresis episodes resolve or are less frequent [6].

According to some authors, children who have enuresis are more likely to have periodic limb movements and OSA. As the etiology of NE is unclear and complicated, it makes the treatment lengthy and frequently accompanied by relapses [7]. NE may occur in children with obstructive SDB due to the decrease in the arousal response [8]. The prevalence of enuresis was reported to be higher in children with suspected SDB [9]; however, some authors suggested that it increases in parallel with OSA severity in girls only [10].

Primary NE can be considered a risk factor for SDB, as it is influenced by OSA and the disruption of urinary hormone regulation such as atrial natriuretic peptide (ANP) and antidiuretic hormone (ADH) [11].

NE is not a benign condition. It may have severe repercussions for the child and their family with negative psychosocial consequences. An enuretic child may be punished with possible emotional or even physical abuse and may experience stress and isolation related to the fear of detection by their peers, with a subsequent lack of self-esteem and poor academic performance [12]. In addition, the behavioral and emotional consequences of sleep disturbances cannot be overlooked, and children with SDB more frequently exhibit daytime symptoms of hyperactivity and ADHD-like symptoms [13]. This highlights the necessity for a detailed investigation of these conditions and the relevant associated factors.

There are many studies that were previously or recently published worldwide that investigate the prevalence and association of NE and SDB. A school-based cross-sectional study conducted in India determined that NE was found in 30.5% (39/128) of children with positive sleep-related breathing disorder (SRBD) [14]. A study that enrolled 1821 subjects was conducted in Greece and proved that the prevalence of enuresis was 7.4% (10/135) in habitual snorers [15]. Another study conducted in Greece that involved 42 children demonstrated that there was a significant association between mouth breathing and NE. A total of 7 out of 42 obstructive sleep apnea–hypopnea syndrome (OSAHS) children had NE [16]. A study conducted in USA involving 480 subjects found that children 6 to 11 years of age with SDB were at a higher risk of having enuresis compared to children without SDB (11.3% vs. 6.3%). In addition, there was a strong association between enuresis and frequent loud snoring [17]. In 2013, a study published in Italy found that primary NE was considered to be a risk factor for sleep disorders such as difficulty in initiating and maintaining sleep, SBD, excessive somnolence, nocturnal hyperhidrosis, and disorders of arousal [1].

Two Egyptian studies investigated the association between NE and SDB with contradictory results. One study conducted in Cairo on 1000 children recruited from general pediatric clinics showed that 153 children (15.3%) had primary NE, with 80 of them being males and 73 being females, with no significant difference between enuretic children with and without SDB [18]. Another study that aimed to detect OSA among school-age children presenting with NE proved that NE was commonly associated with OSA, especially in obese children, where 68.5% of the children with primary NE had OSA [19].

In Saudi Arabia, a cross-sectional study conducted on 640 primary school children determined that the prevalence of enuresis was 16.3% in boys and 13.8% in girls, with an overall prevalence of 15% (CI = 12.2–17.8%) [20]. Another recent Saudi Arabian study conducted in 2021 estimated the prevalence to be as high as 48%, where the prevalence was higher among boys, although the difference did not reach statistical significance [2]. A more recent Saudi Arabian study conducted by Alwadei et al. found that among 1866 children aged 6–12 years who were randomly selected from 20 schools in the city of Al-Kharj, Saudi Arabia, 13% were suffering from a high risk of SDB symptoms (habitual snoring, mouth breathing, witnessed apnea, bedwetting, and being overweight) [21]. Nationwide research on the prevalence and association between SDB and NE among Saudi Arabian children of this age group is still lacking. Hence, we aim to determine whether there is an association between NE and SDB and to assess the prevalence of SDB and NE in primary school children aged 6–12 years in Saudi Arabia.

## 2. Materials and Methods

### 2.1. Study Design

Descriptive quantitative data using cross-sectional observational web-based study.

### 2.2. Study Setting and Participants

This study was conducted among the caregivers of primary school children aged 6–12 years in all Saudi Arabia regions.

### 2.3. Inclusion Criteria

Caregivers of children aged 6–12 years, of both the male and female sexes, living in all administrative districts within Saudi Arabia (Western, Northern, Southern, Central, and Eastern regions), and accepted to complete the survey.

### 2.4. Exclusion Criteria

Children less than 6 years of age or older than 12 years of age, living outside of Saudi Arabia, or did not complete the survey.

### 2.5. Sample Size and Sampling

Using Raosoft sample size calculator (http://www.raosoft.com/samplesize.html, accessed on 1 May 2022), 377 individuals were assessed as the minimum sample size for this study. The study used the following parameters: a standard deviation set at 1.96, an expected response of 50%, a confidence interval of 95%, and an error of no more than 5%. In the sampling strategy, a simple random sample from all Saudi Arabia regions was used. In a trial to achieve a proportional nationwide representative sample and to avoid bias, we tried to double the sample size during the study period from the beginning of May to the end of August 2022 by weighing the age, gender, and population distribution within different geographical Saudi Arabia regions to be as compatible as possible with the demographic data obtained from the Saudi General Authority for Statistics (https://www.stats.gov.sa/en/1007-0, accessed on 1 May 2022) (with accepted ±5% variation). In total, 782 participants took part in the survey, of which 686 (87.7%) met the inclusion criteria.

### 2.6. Data Collection Tools and Processes

The data for this study were gathered through a self-administered online questionnaire in Arabic that takes around 4–5 min to complete. It was created after reviewing the literature and relevant articles with similar aims and validated questionnaires (for example, the Pediatric Sleep Questionnaire and Sleep Disturbances Scale for Children). To assess for content validity, we consulted multidisciplinary specialists in related fields (including pediatric urology and nephrology, sleep medicine, respiratory medicine, and behavior therapy) to authenticate and validate the questions in terms of simplicity, relativity, objectivity, and significance. An independent Arabic language Saudi Arabian academic was invited to proofread the questionnaire before it was translated back into English to correct wording to ensure translation and cultural adaptation.

The questionnaire was piloted on 20 caregivers (excluded from the final analysis) who fulfilled the inclusion criteria, including 4 from each administrative district within Saudi Arabia, to test the comprehensibility of the questionnaire, to detect issues such as wording and question clarity, and to test its validity or any needed modifications, and it was finalized after a series of group discussions. Using Cronbach’s alpha test, the questionnaire’s reliability coefficient was evaluated (α = 0.73). An informed consent form, as a cover page, must be read and accepted by each participant prior to any data collection taking place.

The questionnaire included demographic data, weight and height, and associated comorbidities in addition to the weekly frequencies of snoring symptoms and enuresis, as well as of unrefreshing sleep, that is, “waking up properly in the morning” and “difficulty getting up in the morning” were assessed using Likert-type response scales; “never”, “once”, “twice”, “three to four times”, or “five times or more” per week were further categorized as “never,” “once or twice,” or “three times or more” per week. Children with NE (either primary or secondary) were defined as children who reported NE “once,” “twice,” “three to four times,” or “five times or more” per week [22].

The questionnaire was distributed via different means of communication on the Internet and on social media platforms. In addition, personal communications helped with the rapid distribution of the survey through social networking.

### 2.7. Statistical Analysis

Statistical analysis was performed using R v 3.6.3. Counts and percentages were used to summarize categorical variables, and the mean ± standard deviation was used for continuous variables. Chi-square test or Fisher’s exact test were used to assess the association between categorical variables. Independent samples *t*-test was used to assess the association between continuous variables. Factors associated with NE at the 0.05 level in the univariate analysis were entered into a multivariable regression analysis to assess the independent predictors of NE. Hypothesis testing was performed at a 5% level of significance.

### 2.8. Ethical Consideration

Ethical approval was granted by the Scientific Research Ethics Committee of Najran University in Saudi Arabia, Reference No.: 444-42-5596-DS. Prior to inviting participants to complete an anonymous self-administered questionnaire for data collection, we clearly explained the aims of the research. Participants had the option to continue or stop the survey at any time, with no risk or loss of benefits. The privacy and confidentiality of the participants’ personal information were also protected.

## 3. Results

The questionnaire was completed by 686 respondents (519 mothers, 151 fathers, and 16 guardians). Children who were 6 years of age represented slightly less than one-quarter (22.6%), and those who were ten years of age or older represented 28.9%. A total of 335 children were female (48.8%), while 351 were males (51.2%). About 62.8% of the included children were from the Western and Central regions, and the least (7.7%) were from the Northern region. More than half of the included parents completed university education, and more than one-quarter completed high school. The average weight and height of the included children were 28.5 ± 14.6 kg and 122 ± 24 cm, and more than one-fourth of them (27.6%) were underweight, while more than one-third (36.5%) suffered from being overweight and obese. Most respondents (77.1%) did not report any comorbidities in their children. Asthma, adenotonsillar hypertrophy, and psychological disorders were reported in 16.2%, 15.6%, and 4.8% of children, respectively. NE was reported in 22.3% of the included children. The frequency was <2 times per week in nearly two-thirds of the children (63.4%), and 2+ times per week in 36.6% of the children. The duration of NE was <3 months in 53.6% of the children and 3+ months in 46.4% of the children (Table 1).

**Figure 1:**
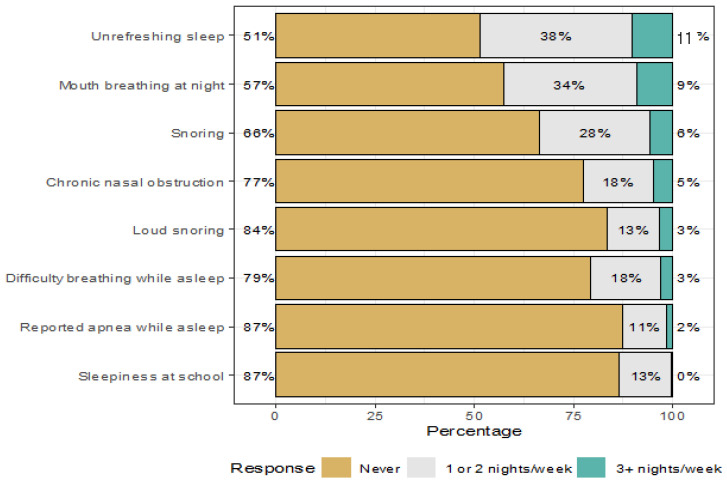
Sleep characteristics of the respondents.

The most common problems reported by the parents were unrefreshing sleep and mouth breathing at night, which were reported to occur once or twice per week in 38% and 34% of the children, and three nights or more per week in 11% and 9%, respectively. Snoring, chronic nasal obstruction, difficulty breathing while asleep, sleepiness at school, and repeated apnea while asleep were reported once or twice per week by 28%, 18%, 18%, 13%, and 11% of the parents, respectively, and three nights or more per week by 6%, 5%, 3%, 0%, and 2% of the parents, respectively. (Figure 1).

The age distribution was significantly different between the respondents with and without NE, with a lower prevalence in children aged 10+ years compared to the remaining age groups (*p* = 0.020). NE was also more prevalent in males than females (25.6% vs. 18.8%, *p* = 0.035). Neither the region nor the parents’ educational level was associated with the prevalence of NE. The average weight and body mass index (BMI) were significantly lower in children with NE (*p* = 0.006 and 0.033, respectively). The BMI categories were associated with the prevalence of NE (*p* < 0.001) (Table 2).

The analysis shows that the sleeping time was associated with NE (*p* = 0.021), as NE was reported in 26.6% of children who slept before 10 PM compared to 19% who slept after 10 PM. Snoring and loud snoring were associated with a higher prevalence of NE (*p* = 0.001 and <0.001, respectively). NE was reported in 28.6% of the patients who snored or loudly snored (57.1%) three or more times per week. These percentages were higher than those observed in the children who never snored (25.9% and 15.5%, respectively). Difficulty breathing while asleep and mouth breathing at night, either 3+ or 1–2 nights/week, were associated with a higher prevalence of NE (*p* < 0.001 and 0.005, respectively). None of the remaining sleep-related characteristics were significantly associated with NE (Table 3).

**Figure 2:**
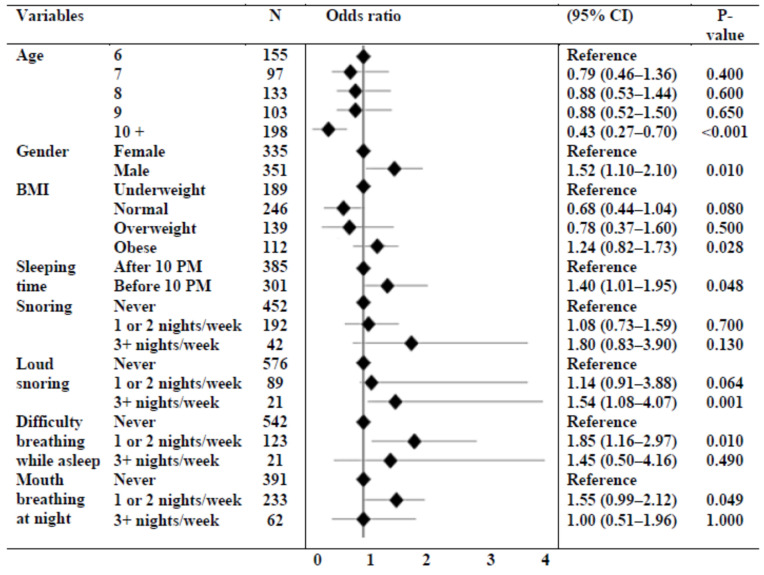
Multivariable regression analysis for factors associated with nocturnal enuresis.

Significant variables associated with NE in the univariate analysis were entered into a multivariable regression analysis to assess the independent predictors of NE. An older age (10+ years) (OR = 0.43, *p* < 0.001) was associated with a lower prevalence of NE. Male gender (OR = 1.52, *p* = 0.010), obesity (OR = 1.24, *p* = 0.028), early sleeping time (OR = 1.40, *p* = 0.048), loud snoring for three or more nights per week (OR = 1.54, *p* = 0.001), difficulty breathing for one or two nights per week (OR = 1.85, *p* = 0.010), and mouth breathing at night for one or two nights per week (OR = 1.55, *p* = 0.049) were associated with higher odds of NE (Figure 2).

## 4. Discussion

### 4.1. Association between NE and SDB

In this study, NE was reported in 22.3% of the included children, with a frequency of two or more times per week in 36.6% of the children. Sleeping time was associated with NE (*p* = 0.021), as NE was reported in 26.6% of the respondents who slept before 10 PM compared to 19% who slept after 10 PM. It was also more prevalent in males than in females (*p* = 0.035).

There was evidence of an association between NE and SDB. In a large population-based study using a full polysomnography to examine the association between parasomnias and SDB, Hispanic and Caucasian children aged 6 to 11 years with SDB were more likely to have enuresis (11.3% vs. 6.3%, *p* < 0.08) than children without SDB [17]. Furthermore, in their study to determine the association between primary NE and habitual snoring in children with OSAHS, Sakellaropoulou et al. reported that 7/42 (16.7%) of Greek children aged 3.5 to 14.5 years who had OSAHS presented with NE, and habitual snorers were at a greater risk of having NE than non-snorers (OR: 1.33, 95% CI: 1.05–1.68). However, the prevalence of enuresis was not related to the severity of OSAHS, as expressed via the apnea hypopnea index (AHI) (*p* = 0.70) [15].

Based on the present study, the multivariable regression analysis showed that loud snoring for three or more nights per week and difficulty breathing for one or two nights per week had significant association with NE (*p* = 0.001 and *p* = 0.010, respectively). Mouth breathing at night (one or two nights per week) was also associated with higher odds of NE (OR = 1.55, *p* = 0.049).

Our study discovered a significant frequency of NE among SDB. We found an association between NE and sleeping time, trouble breathing, and mouth breathing at night. On the other hand, Su et al. examined the prevalence and correlation of NE among primary school Chinese children aged 6–11 years and its relation to OSA. They found that boys had a significantly greater prevalence of NE than girls (OR: 2.57, 95% CI: 1.83–3.61, *p* < 0.001); however, no difference was detected in the prevalence of NE between children with and without OSA (9.7% vs. 8.8%; *p* = 0.725) [10]. Prior research suggests that children with NE sleep more deeply and are more difficult to arouse (high arousal threshold), which prevents arousals when the bladder is full [16,23]. However, these children may experience higher levels of daytime sleepiness that might be explained by the episodes of bedwetting and attempts to keep the child dry during the night resulting in sleep fragmentation, which leads to an increased arousal threshold, which, in turn, leads to the failure to respond to full-bladder signals, and the result is additional bedwetting with subsequent clinical implications for enuresis management [24].

Using regression analysis, we were unable to demonstrate a relationship between snoring and NE, while loud snoring was significantly associated. Earlier studies indicated a positive relationship between frequent snoring and NE [16,23]. This difference might be attributed to different definitions of NE. In addition, it is possible that it is connected to varied study designs and sample sizes.

### 4.2. Prevalence of NE

Our study reports a prevalence of NE among primary school children in Saudi Arabia that is 22.3%. This finding is less than the results reported by Alhifthy et al. in their cross-sectional study among children aged 5–18 years in the Eastern region in Saudi Arabia that showed a high prevalence of around 48.3%. [2]. However, in their cross-sectional questionnaire-based study in Jazan, Saudi Arabia, Sherah et al. found a substantially greater prevalence in their region, with 76.4% of children aged 5 to 12 years having NE, including 52.6% corresponding to the primary type of NE. They found that low school performance, pinworm infestation, and low educational qualification of the respective fathers were identified as significant risk factors [25]. Possible selection bias and the use of online questionnaires and self-reported data are major limitations of these studies. On the other hand, a cross-sectional study conducted by Alshahrani et al. in primary health care centers of family and community medicine in Riyadh, Saudi Arabia using a personal interview among children aged 5 to 12 years reported a lower prevalence rate of 18.5% for NE [26]. It was also reported that there was a much lower prevalence of NE among school children in Turkey (12.4%), Italy (7.2%), and Thailand (3.9%) [27,28,29].

According to a systemic review to determine the prevalence of enuresis and its associated variables among Iranian children, the overall prevalence of enuresis was estimated to be 11.01% [30]. The differences in prevalence could be attributed to differences in the sample size, sampling method, age range of the cohort that was surveyed, the method used to gather the data (self-administered questionnaire vs. direct interview), and the definition of NE, which could be based on the Diagnostic and Statistical Manual (DSM-IV) or the International Classification of Diseases (ICD-10) criteria. The variations in prevalence between the countries and regions could also be affected by medical, psychological, socioeconomic, cultural, and racial factors [31,32].

### 4.3. Risk Factors for NE

In terms of the risk factors for NE in the examined children, our study reveals an association between NE and child age, since the odds of prevalence was lower as the ages of the children increased (OR = 0.43, *p* < 0.001). This is consistent with other investigations that found that NE prevalence rates decline with age [32,33,34]. It was proposed that, after the age of 7 years, NE can be spontaneously cured at a rate of 15% annually; however, some patients remain enuretic even beyond the age of 16 years [35]. Generally, NE is considered a multifactorial disease that is associated with a complex interaction of somatic, environmental, and psychosocial factors. Numerous potential causes of NE were proposed, including hereditary aberration, a delayed maturity of the neural system that regulates bladder function, a delayed development of the bladder size and function, sleep-arousal disorder, neuropsychological disorders, and a reduced release of ADH at night [36,37,38]. Researchers suggested other variables that may contribute to the development of NE in childhood and adolescence, including hypercalcemia, low vitamin B12 levels, migraines, frequent habitual snoring, and changes to the brain’s microstructure [15,39,40,41,42]. These variations may be explained by a different choice of age groups or by a combination of racial, cultural, social, and medical variables. Moreover, little is known about the specific function of brain regions in the context of NE. Recently, functional neuroimaging studies provided a noninvasive approach to investigate the functional and structural changes associated with NE [38].

In our study, we also found a substantial gender difference in the prevalence of NE, with males having a higher frequency and prevalence than females (25.6% vs. 18.8%, *p* = 0.035) (OR = 1.52, *p* = 0.010), which is consistent with the findings of other research [32,43,44]. A large study that involved nearly 20,000 primary school children in Japan determined that unrefreshing sleep and snoring were associated with a higher prevalence of NE in boys than in girls [22]. These results seem to be modulated by a variety of developmentally controlled variables. First, there may be a difference in the renal sensitivity to vasopressin between boys and girls (significantly higher in boys), and vasopressin-mediated effects on renal and vascular targets are more pronounced in boys than in girls, which may have an impact on the urine volume [45]. Second, girls have enhanced bladder control-related brain development [46]. Finally, females often communicate their need for bladder evacuation earlier, which causes them to begin toilet training earlier [46]. However, several studies, notably, those conducted in China and India, did not show significant differences in this area [47,48]. Studies from Turkey, Sanandaj (Iran), and Aden (Yemen), among others, revealed that girls are more likely than boys to experience NE [49,50,51].

No significant relationship between the parental educational level and the prevalence of NE was discovered in the present research, which followed the same trend as the findings of a study by Gunes et al. in Turkey [52]. A possible explanation may be related to the fact that in Saudi Arabian community and culture, Saudi Arabian parents with a low education level do not necessarily have a poor socioeconomic status due to high economic development within the country. According to several works of research, NE is more common in families with lower paternal education levels than higher maternal education levels [53]. Other research has also shown a connection between NE prevalence and lower parental education levels [27]. Van Hoecke et al. identified low socioeconomic status, including low education level, as a significant risk factor for psychopathology that may explain the variance in NE prevalence [54].

Our study reveals an association between obesity and NE, where obesity is associated with higher odds of NE (OR = 1.24, *p* = 0.028). Similar studies demonstrated the association between obesity and NE, with obesity raising the risk of the condition [55,56]. Ma et al. discussed possible explanations for such an association, including the following: obese children tend to consume an unhealthy diet that may overwhelm their functional bladder capacity; obesity exposes the pelvic floor to elevated intra-abdominal and intra-vesical pressure, which reduces the functional bladder capacity; obesity and enuresis are closely related to psychological factors; obesity is associated with hyperglycemia, which can cause diuresis; and obesity is associated with SDB, which is directly related to NE [57]. Contrary to our findings, other studies have reported that obesity and NE are not associated [58,59]. To confirm or deny such a relationship, further research should be conducted and compared to the general population.

### 4.4. Limitations of the Study

This study has several limitations. First, the data were collected via self-administered questionnaires, which primarily relied on the patients’ honesty and subjective opinions, so this may result in inaccurate answers with possible recall bias of self-reported data and social desirability bias. Second, causal inferences are difficult with a cross-sectional design. Third, this study targets children aged 6–12 years, so the generalizability of our results is limited. Fourth, there is a potential selection bias when using a web-based survey (being accessible to web-users only with limited participation of individuals who are poor or less likely to use technology (e.g., social media) and possible over-representation of more interested individuals). Fifth, we utilized a questionnaire created by a modification of previously used tools on NE and SDB; however, the validation of the modified questionnaire is a concern. Sixth, residual confounding caused by unmeasured covariates possibly occurred due to possible contributors such as the family’s circumstances and the child’s emotional and behavioral function, nutritional status, and exposure to stress. Finally, the children were not evaluated via polysomnography, in addition to a lack of clinical confirmation of the condition.

## 5. Conclusions

Our study reveals that 22.3% of primary school children in Saudi Arabia reported to be suffering from NE. The prevalence was higher in males than in females, whereas it was lower as the children aged. SDB is a common problem among children with NE. The exact mechanism that links SDB to the increase in the risk of NE is unknown. Male gender, obesity, early sleeping time, loud snoring, difficulty breathing, and mouth breathing at night are potential independent risk factors of NE in school-age children.

Integrating community-based intervention programs to provide counseling and supportive care for parents and children with NE, together with in-service training programs for healthcare professionals, can make the management of NE less difficult and decrease the social, psychological, and financial implications for the families and their children.

## Figures and Tables

**Table 1:** Descriptive statistics for the study sample.

Variables		*n* = 686 (%)
**Age** (years)	6	155 (22.6)
	7	97 (14.1)
	8	133 (19.4)
	9	103 (15.0)
	10+	198 (28.9)
**Gender**	Female	335 (48.8)
	Male	351 (51.2)
**Relation**	Mother	519 (75.7)
	Father	151 (22.0)
	Guardian	16 (2.3)
**Region**	Western	226 (32.9)
	Central	205 (29.9)
	Eastern	103 (15.0)
	Southern	99 (14.4)
	Northern	53 (7.7)
**Average parental education level**	Illiterate	7 (1.0)
	Primary school	40 (5.8)
	High school	198 (28.9)
	Bachelor’s degree	396 (57.7)
	Postgraduate degree	45 (6.6)
**Weight** (kg)		28.5 ± 14.6
**Height** (cm)		122.0 ± 24.0
**BMI** (kg/m^2^)		20.0 ± 8.2
**BMI category**	Underweight (<18.5)	189 (27.6)
	Normal (18.5–24.9)	246 (35.9)
	Overweight (25–29.9)	139 (20.2)
	Obese (>30)	112 (16.3)
**Comorbidities ^1^**	None	529 (77.1)
	Asthma	111 (16.2)
	Adenotonsillar hypertrophy	107 (15.6)
	Other pulmonary disorders	55 (8.0)
	Psychological disorders	33 (4.8)
	Cardiac disorders	24 (3.5)
	Other	13 (1.9)
**Nocturnal enuresis**		153 (22.3)
**Frequency of nocturnal enuresis**	<2 times/week	97 (63.4)
	2+ times/week	56 (36.6)
**Duration of nocturnal enuresis**	<3 months	82 (53.6)
	3+ months	71 (46.4)

Counts and percentages were used to summarize categorical variables and mean ± SD for continuous variables. **^1^** Some children have more than one comorbid condition.

**Table 2:** Sociodemographic characteristics associated with nocturnal enuresis.

Sociodemographic Characteristics		Nocturnal Enuresis		*p*-Value
		Yes *n* = 153 (%)	No *n* = 533 (%)	
**Age** (years)	6	39 (25.2)	116 (74.8)	0.020 *
	7	22 (22.7)	75 (77.3)	
	8	35 (26.3)	98 (73.7)	
	9	29 (28.2)	74 (71.8)	
	10+	28 (14.1)	170 (85.9)	
**Gender**	Female	63 (18.8)	272 (81.2)	0.035 *
	Male	90 (25.6)	261 (74.4)	
**Region**	Western	51 (22.6)	175 (77.4)	0.097
	Central	45 (22.0)	160 (78.0)	
	Eastern	30 (29.1)	73 (70.9)	
	Southern	22 (22.2)	77 (77.8)	
	Northern	5 (9.4)	48 (90.6)	
**Average parental education level**	Illiterate	2 (28.6)	5 (71.4)	0.414
	Primary school	10 (25.0)	30 (75.0)	
	High school	41 (20.7)	157 (79.3)	
	Bachelor’s degree	85 (21.5)	311 (78.5)	
	Postgraduate degree	15 (33.3)	30 (66.7)	
**Weight** (kg)		26.4 ± 13.2	30.2 ± 15.5	0.006 *
**Height** (cm)		121.0 ± 22.1	124.0 ± 25.3	0.184
**BMI** (kg/m^2^)		19.2 ± 8.0	20.8 ± 8.2	0.033 *
**BMI category**	Underweight	31 (16.4)	158 (83.6)	<0.001 *
	Normal	48 (19.5)	198 (80.5)	
	Overweight	29 (20.9)	110 (79.1)	
	Obese	45 (40.2)	67 (59.8)	

Values presented as number and percentage were analyzed using Chi-square test or Fisher’s exact test. Values presented as mean ± SD were analyzed using independent samples *t*-test. * Significant.

**Table 3:** Association between sleep-related characteristics and nocturnal enuresis.

Sleep-Related Characteristics		Total *n* = 686 (%)	Nocturnal Enuresis		*p*-Value
			Yes *n* = 153 (%)	No *n* = 533 (%)	
**Sleep duration**	>9 h	276 (40.2)	64 (44.6)	212 (55.4)	0.708
	9 h or less	410 (59.8)	89 (41.7)	321 (58.3)	
**Sleeping time**	After 10 PM	385 (56.1)	73 (19.0)	312 (81.0)	0.021 *
	Before 10 PM	301 (43.9)	80 (26.6)	221(73.4)	
**Snoring**	Never	452 (66.0)	117 (25.9)	335 (74.1)	0.001 *
	1 or 2 nights/week	192 (28.0)	24 (12.5)	168 (87.5)	
	3+ nights/week	42 (6.0)	12 (28.6)	30 (71.4)	
**Loud snoring**	Never	576 (84.0)	89 (15.5)	487 (84.5)	<0.001 *
	1 or 2 nights/week	89 (13.0)	52 (58.4)	37 (41.6)	
	3+ nights/week	21 (3.0)	12 (57.1)	9 (42.9)	
**Unrefreshing sleep**	Never	350 (51.0)	75 (21.4)	275 (78.6)	0.780
	1 or 2 nights/week	260 (38.0)	59 (22.7)	201 (77.3)	
	3+ nights/week	76 (11.0)	19 (25.0)	57 (75.0)	
**Reported apnea** **while asleep**	Never	597 (87.0)	128 (21.4)	469 (78.6)	0.372
	1 or 2 nights/week	75 (11.0)	21 (28.0)	54 (72.0)	
	3+ nights/week	14 (2.0)	4 (28.6)	10 (71.4)	
**Difficulty breathing while asleep**	Never	542 (79.0)	83 (15.3)	459 (84.7)	<0.001 *
	1 or 2 nights/week	123 (18.0)	63 (51.2)	60 (48.8)	
	3+ nights/week	21 (3.0)	7 (33.3)	14 (66.7)	
**Sleepiness at school**	Never	597 (87.0)	129 (21.6)	468 (78.4)	0.275
	1 or 2 nights/week	89 (13.0)	24 (27.0)	65 (73.0)	
	3+ nights/week	0 (0.0)	0 (0.0)	0 (0.0)	
**Mouth breathing at night**	Never	391 (57.0)	70 (17.9)	321 (82.1)	0.005 *
	1 or 2 nights/week	233 (34.0)	64 (27.5)	169 (72.5)	
	3+ nights/week	62 (9.0)	19 (30.6)	43 (69.4)	
**Chronic nasal obstruction**	Never	528 (77.0)	113 (21.4)	415 (78.6)	0.540
	1 or 2 nights/week	123 (18.0)	32 (26.0)	91 (74.0)	
	3+ nights/week	35 (5.0)	8 (22.9)	27 (77.1)	

Values presented as number and percentage were analyzed using Chi-square test or Fisher exact test. * Significant.

## Data Availability

The datasets used and analyzed in the current study are available from the corresponding authors upon reasonable request. The confidentiality and security of the data and materials were ensured throughout all stages of the study.

## Abbreviations

ADH	Antidiuretic hormone
AHI	Apnea hypopnea index
ANP	Atrial natriuretic peptide
BMI	Body mass index
CI	Confidence interval
DSM	Diagnostic and Statistical Manual
ICD	International Classification of Diseases
NE	Nocturnal enuresis
OR	Odds ratio
OSA	Obstructive sleep apnea
OSAHS	Obstructive sleep apnea–hypopnea syndrome
SDB	Sleep-disordered breathing
SRBD	Sleep-related breathing disorder
